# Editorial: Unraveling circRNAs and miRNAs: key regulators in immune-related diseases

**DOI:** 10.3389/fimmu.2025.1739926

**Published:** 2025-11-28

**Authors:** Mohamad S. Hakim, Aqsa Ikram, Mounir M. Salem-Bekhit, Zulqarnain Baloch

**Affiliations:** 1 Department of Biology and Immunology, College of Medicine, Qassim University, Buraydah, Saudi Arabia; 2 Institute of Molecular Biology and Biotechnology, University of Lahore, Lahore, Pakistan; 3 Department of Pharmaceutics, College of Pharmacy, King Saud University, Riyadh, Saudi Arabia; 4 Faculty of Life Science and Technology, Kunming University of Science and Technology, Kunming, China

**Keywords:** circRNAs, miRNAs, non-coding RNA, immune-associated diseases, systems biology

**Figure 1:**
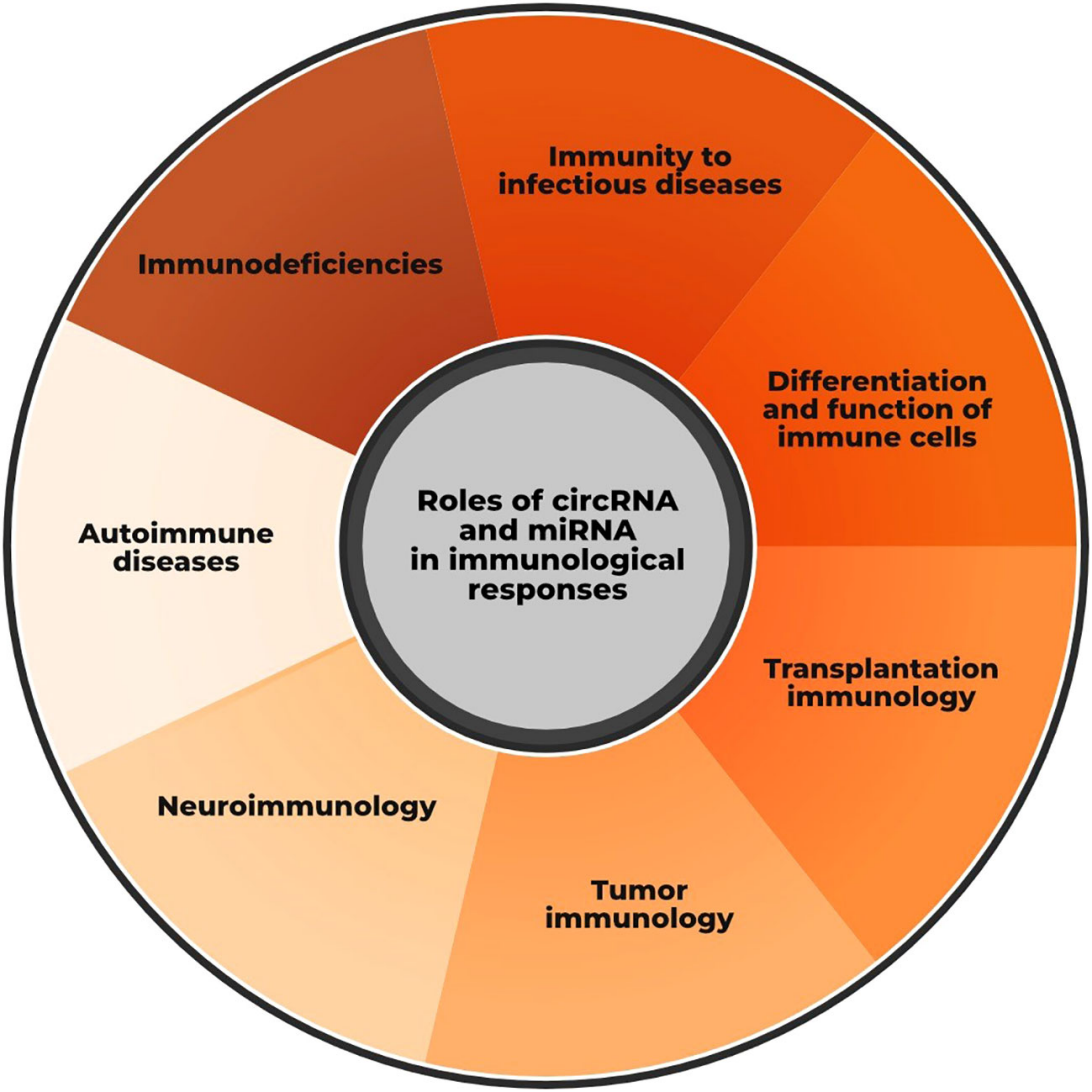
Roles of circular RNAs (circRNAs) and microRNAs (miRNAs) in regulating immune responses —such as those in transplantation, tumor, and neuroimmunology— and in the pathogenesis of immune-related diseases.

In recent years, there has been increasing interest in the field of immunology in unraveling the role of non-coding RNAs (ncRNAs), especially circular RNA (circRNAs) and microRNA (miRNAs), in the development and pathogenesis of various immune-associated diseases (**Figure 1**). In contrast to RNA that encodes for functional proteins within the cells, these molecules are not translated into proteins in the ribosome, yet they play essential roles in regulating gene expression and homeostasis, as well as in controlling the strength and magnitude of immune responses. CircRNAs and miRNAs have recently been identified as key players in modulating the activation, proliferation, and differentiation of various components of the immune response, including macrophages, B cells, T cells, and others. Furthermore, increasing evidence shows that they can be utilized in diagnosis, as biomarkers, in monitoring the effectiveness of immunotherapy, and in predicting clinical outcomes in various immune-associated diseases, including autoimmunity, chronic inflammation, and immune-related cancers. Nevertheless, despite strong evidence of their functional role in immune-associated diseases, many in-depth aspects of how circRNAs and miRNAs operate within the immune system, particularly how they interact with each other within molecular and immunological regulatory networks, remain poorly understood.

This Research Topic aims to further delineate the contribution and mechanisms of action of circRNAs and miRNAs in immune-associated disease. Our focus includes studies about the biogenesis and expression dynamics of these non-coding RNA in various physiological and pathological conditions, and how these molecules mediate alterations at the molecular level that ultimately affect the functionality of the immune system. This Research Topic also explores the roles of microRNAs and circRNAs in regulating specific gene targets involved in immune signaling networks. The approach used involves integrating data from various advanced technologies, such as RNA sequencing, transcriptomic analysis, and regulatory network modeling using systems biology approaches. By utilizing these approaches, it is expected to have a more comprehensive insight into the causal relationship between non-coding RNA expression and the onset of immunological disorders.

**Figure 2:**
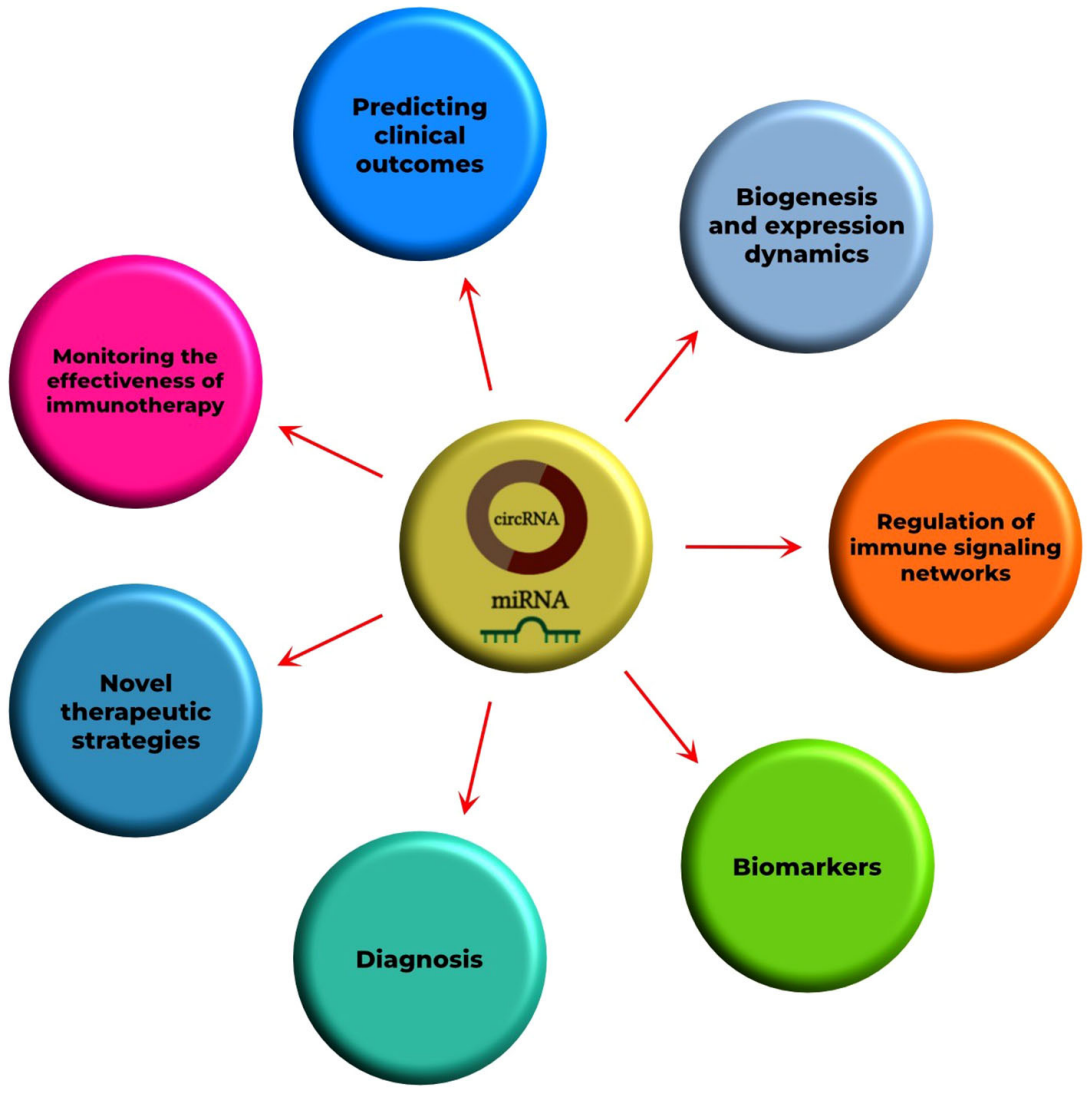
Roles of circRNAs and miRNAs in immune-related diseases. It is essential to comprehend the fundamental concepts of circRNAs and miRNAs, including their biogenesis and expression dynamics in various physiological and pathological conditions, as well as their contribution to regulating specific gene targets involved in immune signaling networks. Our understanding would serve as the basis for the clinical aspects of immune-related diseases, such as the development of biomarkers, diagnostic tools, and novel therapeutic strategies, as well as monitoring the effectiveness of immunotherapy and predicting clinical outcomes.

Overall, this Research Topic holds potential for the development of new diagnostic and therapeutic strategies for immune-associated diseases that currently lack effective treatments. The identification of key molecular networks mediated by circRNAs and miRNAs could be exploited to develop personalized therapy guided by patients’ molecular profiles. In addition, this Research Topic can lay the groundwork for the identification of novel biomarkers that are not only utilized in clinical practice but also enhance our understanding of the fundamental biology of the human immune system (**Figure 2**). Bridging the knowledge gap in this field represents a strategic step toward overcoming the clinical challenges posed by the complexity of modern immune-related diseases, including those that are rare or resistant to current conventional therapies.

This Research Topic consists of five review papers and four original research articles. In one of the article, Yang et al. discussed the role of circRNAs in inflammatory bowel diseases (IBD). They highlighted dysregulated in circRNA expression in the blood, exosomes, cells, and colonic tissues of IBD patients. They also discussed three potential mechanisms of circRNAs in IBD pathogenesis, such as acting as “sponges” for miRNAs, interacting with RNA-binding proteins (RBPs) to modulate RNA stability, and post-transcriptional gene expression regulation. Importantly, this review also highlights how circRNAs influence molecular pathways that drive the progression of colitis-associated cancers (CAC). Finally, they discussed the emerging roles of therapeutic targeting of circRNAs in IBD, including RNA interference-mediated circRNA knockdown and viral vector-mediated circRNA overexpression.

Fatima et al. explored the role of RNA modifications in rheumatoid arthritis (RA). The authors reviewed current knowledge on how post-transcriptional modifications, such as N6-methyladenosine (m6A), influence gene expression and immune responses in the synovial tissue of RA patients. They also explored how these modifications may disrupt synovial homeostasis and contribute to RA pathogenesis. The review highlighted the need for further research to understand the complex interactions between RNA modifications and immune regulation in RA, which could lead to novel therapeutic strategies.

Chen et al. deeply discussed the role of circRNAs as novel strategies to treat breast cancers. They first mentioned the fundamental biology of circRNAs, including the role of circRNAs as microRNA sponges, regulators of gene expression and immune responses, as well as in protein translation. Subsequently, they discussed how circRNAs are involved in the development, pathogenesis, invasion, and metastasis of breast cancers. For example, circRNAs play a regulatory role in the regeneration and proliferation of breast cancer stem cells. They also influence the breast tumor immune microenvironment necessary for cancer progression. Thus, circRNAs can be utilized as potential biomarkers for diagnosis, staging, and prognosis, as well as promising therapeutic targets for breast cancer. CircRNAs also affect the resistance of breast cancer to chemotherapy.

You et al. thoroughly discussed the crosstalk between ncRNAs and the ubiquitin ligases in the progression of cardiovascular diseases. The ubiquitin–proteasome system (UPS) is the primary non-lysosomal pathway for targeted protein degradation in eukaryotic cells. miRNAs can exert either positive or negative regulatory effects on the process of ubiquitination, influencing protein stability. The interaction between the UPS and ncRNAs plays a crucial role in modulating the pathological mechanisms underlying cardiac (e.g., myocardial infarction, cardiomyopathy, myocarditis, and heart failure) and vascular diseases (e.g., atherosclerosis and stroke).

The last review paper by Urzi et al. comprehensively synthesized the emerging role of circRNAs in muscular immune-related diseases. They highlighted the role of circRNAs in various physiological and pathological processes in both skeletal muscle and immune cells, as well as identified research gap in this field to inform future studies. They also discussed promising strategies to engineer circRNAs as therapeutic agents in immune-associated muscle diseases.

Živanović et al. aimed to identify specific miRNA expression patterns in sinonasal tissue that could aid in diagnosing granulomatosis with polyangiitis (GPA), an autoimmune vasculitis. They conducted a comparative analysis of miRNA expression in sinonasal biopsies from patients with GPA and control subjects using high-throughput sequencing techniques. They discovered distinct miRNA signatures associated with GPA, highlighting their potential as diagnostic biomarkers. These findings suggest that miRNA profiling of sinonasal tissue could enhance diagnostic accuracy for GPA and improve patient management.

Mitsuyama et al. evaluated the expression of mRNA and miRNA in acute respiratory distress syndrome (ARDS) patients and explored the pathogenesis of ARDS after performing integration analysis. In this prospective, single-center observational study, peripheral blood samples were obtained within 24 hours of hospital admission. mRNA and miRNA sequencing were conducted on whole blood collected from both ARDS patients (n=34) and healthy controls (n=15). The study revealed that, in comparison to healthy donors, ARDS patients showed upregulation of 1,233 mRNAs and 6 miRNAs, along with downregulation of 1,580 mRNAs and 13 miRNAs. These molecular alterations triggered activation of the PD-1/PD-L1 signaling axis and inhibited the Th2 immune response, resulting in dysfunctional T cells observed in ARDS patients. This study highlights the potential roles of multiple miRNAs in the development and progression of ARDS.

Sun et al. identified key diagnostic lncRNAs in osteonecrosis of the femoral head (ONFH) with metabolic syndrome (MetS) and constructed an immune-related competitive endogenous RNA (ceRNA) network. Plasma RNA profiles from ONFH patients and controls were analyzed and integrated with a MetS dataset. Bioinformatics methods, including weighted gene co-expression network analysis (WGCNA), protein-protein interaction (PPI) analysis, and random forest (RF) algorithms identified hub genes and related lncRNAs. A diagnostic model based on five lncRNAs showed strong performance (AUC > 0.7). These lncRNAs may serve as noninvasive biomarkers for early ONFH detection in MetS populations.

Yan et al. evaluated the role of ZC3H13 in m6A methylation and its impact on the tumor microenvironment (TME) of esophageal squamous cell carcinoma (ESCC). Bioinformatics and experimental analyses in patient samples and xenograft models were used to assess ZC3H13 expression, m6A levels, cytokine regulation, and macrophage infiltration. Results showed that high ZC3H13 expression enhanced m6A modification, stabilized CXCL8 mRNA, and promoted M2 macrophage polarization via the CXCL8-CXCR2 axis. Silencing ZC3H13 reduced tumor growth, CCL5/CXCL8 expression, and M2 macrophage infiltration. Overall, ZC3H13 drives ESCC progression by linking m6A modification to immune regulation in the TME.

As a closing remark, this is an overview of the articles published under this Research Topic, which extensively discuss various immune-associated diseases, including IBDs, RA, CVDs, and breast cancer. We hope that this Research Topic can make a significant contribution to this continually evolving field, although translating these findings to clinical practice presents a considerable challenge.

